# Epithelial-Mesenchymal Interaction in Hair Regeneration and Skin Wound Healing

**DOI:** 10.3389/fmed.2022.863786

**Published:** 2022-04-14

**Authors:** Mei-Qi Mao, Jing Jing, Yu-Jie Miao, Zhong-Fa Lv

**Affiliations:** Department of Dermatology, Second Affiliated Hospital, Zhejiang University School of Medicine, Hangzhou, China

**Keywords:** skin, skin appendages, dermal papilla, epithelial-mesenchymal interactions, wound healing, hair follicle growth cycle

## Abstract

Interactions between epithelial and mesenchymal cells influence hair follicles (HFs) during embryonic development and skin regeneration following injury. Exchanging soluble molecules, altering key pathways, and extracellular matrix signal transduction are all part of the interplay between epithelial and mesenchymal cells. In brief, the mesenchyme contains dermal papilla cells, while the hair matrix cells and outer root sheath represent the epithelial cells. This study summarizes typical epithelial–mesenchymal signaling molecules and extracellular components under the control of follicular stem cells, aiming to broaden our current understanding of epithelial–mesenchymal interaction mechanisms in HF regeneration and skin wound healing.

## Introduction

Hair follicles (HFs) consist of the infundibulum, isthmus, and hair bulb. The hair bulb is located in the thickened base of the hair root and consists of an epithelium-derived matrix wrapped around a mesenchymal cell-derived dermal papilla (DP), which contains DP cells, endothelial vascular cells, and extracellular matrix (ECM). Cell–cell contacts, cell–matrix interactions, and tissue–neural interplay are all controlled by epithelial–mesenchymal interactions (EMIs), which also incorporate morphogens, cell adhesion factors (proteoglycans, etc.), growth factors, ECM molecules, hormones, cytokines, enzymes, and specific pharmacologically relevant molecules (retinoid, etc.) and their receptors (1).

Sonic hedgehog (SHH), wingless (Wnt), bone morphogenetic protein (BMP), fibroblast growth factor (FGF), their receptors, and other pathways are linked to embryonic HF development, the hair cycle, and skin wound healing (2). Other underlying molecular families associated with HF morphogenesis are the transforming growth factor-beta (TGF-β) family and neurotrophic proteins (3, 4). In HFs, DPs can secrete components that act on the peripheral matrix, such as epidermal growth factor (EGF), FGF, hepatocyte growth factor (HGF), insulin-like growth factor-I (IGF-I), keratinocyte growth factor (KGF or FGF-7), TGF-β, basic FGF (bFGF or FGF-2), and interleukins (IL-1, etc.) (5). DP ensures and regulates the hair growth and hair cycle in order.

Through EMI between DPs and epithelial cells, HFs participate in postinjury skin wound healing. In patients with extensive skin burns, transplanting HF progenitor cells enhances angiogenesis, regulates the inflammatory response, speeds wound healing, and improves the physiological function of skin regeneration. HF progenitor cell transplantation is an innovative technique and could be a new therapeutic option for long-term unhealed wounds (6).

## EMI in HF Morphogenesis and Cyclic Regeneration

### HF Morphogenesis

**Figure 1:**
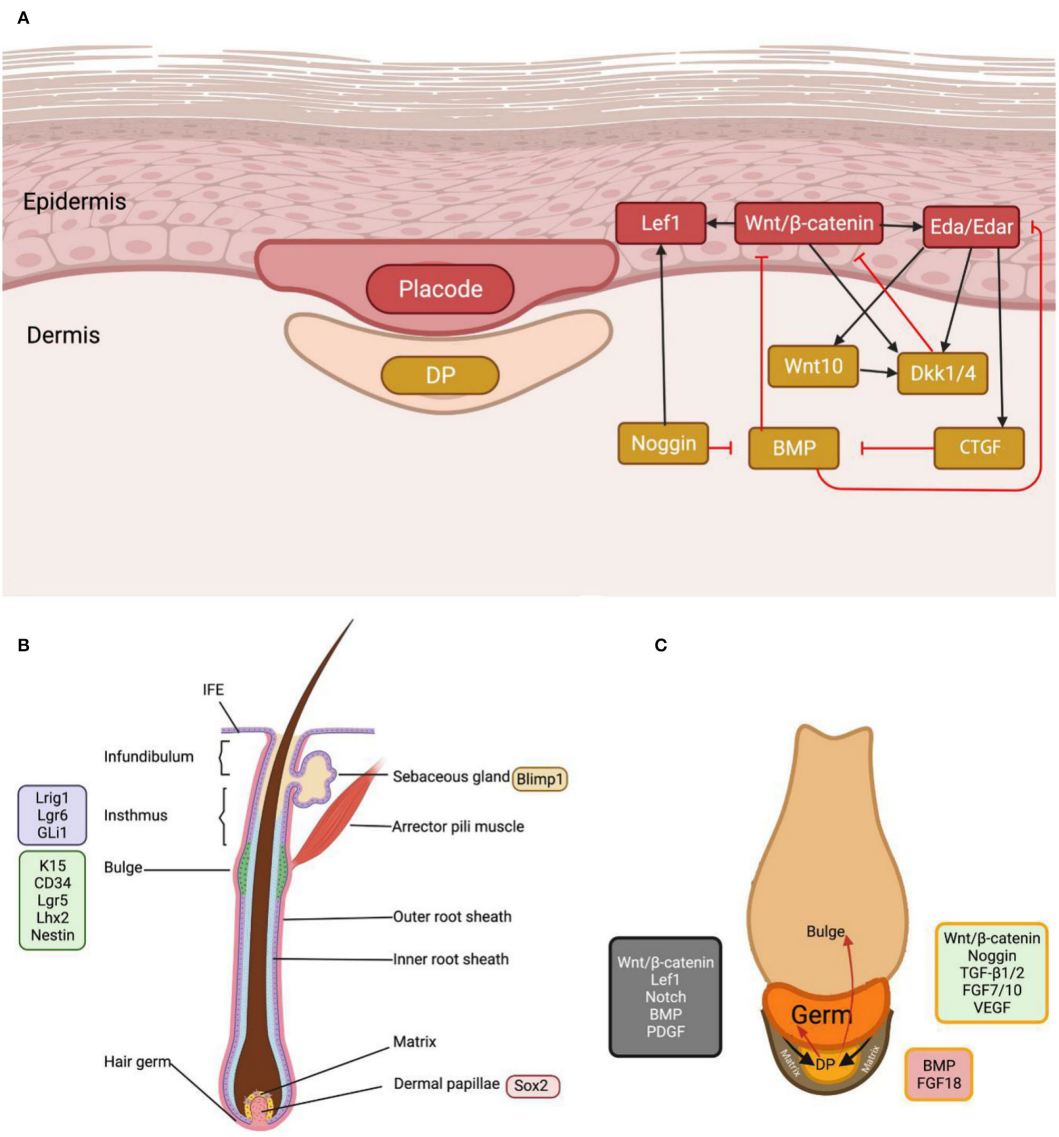
The morphogenesis and cycling of the hair follicle. **(A)** As the earliest and paramount signal in the first hair wave, Wnt induces placode and dermal condensate formation and intrigues complex inter-signal dynamics. **(B)** In mature hair follicles, stem cells at different sites can be characterized by specific markers. **(C)** EMI interaction occurs between DP, hair germ and its progeny, and hair matrix, during hair anagen initiation. The green color in the figure represents DP signals that initiate the anagen, the red color inhibits the hair follicle cycle, and the gray color represents the matrix signals. BMP and FGF18 suppress hair stem cells, whereas Wnt and FGF7/10 activate them. CTGF, connective tissue growth factor; Eda, ectodysplasin A; Edar, ectodysplasin A receptor; DKK, Dickkopf; Gli1, GLI family zinc finger 1; Lef1, lymphoid enhancer-binding factor 1; Lgr, leucine-rich repeat-containing G-protein coupled receptor; Lhx2, LIM homeobox 2; Lrig1, leucine-rich repeats and immunoglobulin-like domains protein 1.

Hair follicle morphogenesis is the climax of a series of EMI through coordinated epidermal–mesenchymal signaling and gradual tissue remodeling, with stem cell populations evolving into a complete HF structure (7, 8). Due to the availability of mouse specimens, mouse models play a key role in EMI research during HF morphogenesis and cycle (9). Initially, the dermis emits the first dermal signal, which stimulates the production of epidermal placodes. The placode is the initial hair structure, a concavity of the epidermis descending into the dermis. Subsequently, the placode delivers epidermal impulses to the dermal cells beneath the epidermis, leading to the formation of dermal condensate (DC) (7, 8). Following the DC's second dermal signal, proliferative epithelial cells shape pegs and downwards (Figure 1A). HF stem cells (HFSCs) stimulate the subsequent downward expansion of hair pegs, eventually forming an integral HF.

### HF Cyclic Regeneration

Bulge HFSCs and mesenchymal DPs act as progenitor cells for the HF epidermal and dermal layers, respectively (7). In the late telogen phase of humans and mice, DP activates bulge HFSCs, creating germ cells that continue to build the hair matrix, resulting in the production of the inner root sheath and the hair sheath (10).

Dermal papilla numbers are stable during HF cycles but drastically decrease in patients with androgenetic alopecia (11). Maintenance of the quantity and function of DP cells is required for healthy hair. In normal conditions, HF dermal stem cell (hfDSC) progeny supply lower DP cells and diverge toward the dermal sheath, but with injury, cell loss, senescence, and HF hypertrophy, they are transported to the upper DP, which initiates significant HF regeneration (12). During the anagen phase, hfDSCs replenish the DP and dermal sheath and exit the DP into the dermal cup during the catagen phase. Then, they enter a quiescent state or undergo apoptosis. Accordingly, to maintain the HF cycle, a balance between hfDSC reduction (differentiation and withdrawal from the hfDSC niche) and increase is critical (13).

The dermal papilla is dependent on EMI to induce HF regeneration, and the function of DP is inextricably tied to the progenitors of the epithelial matrix that surrounds it (14). The hair matrix is located in the proliferative zone of the hair bulb and consists of epithelial stem cells and transient amplifying cells. DP awakens the transient amplifying cells in anagen, enabling hair germs to migrate down with the DP, similar to how epithelial stem cells behave during embryonic HF development (Figure 1C) (8). In late anagen and catagen, DP loses contact with the hair matrix and ascends below the lower bulge as the hair sheath shortens. Moreover, the hair matrix transforms into secondary hair germs. In the next cycle, the secondary hair germ encloses the DP to form the newly generated hair matrix (15, 16).

### EMI Signals Modulate HF Morphogenesis and Cycling

#### Wnt, SHH, and BMP Pathway

The epidermal NF-κB, Wnt/β-catenin, and SHH/patched pathways control HF development. In the mesenchyme, BMP signaling takes a predominant position, promotes HF regeneration, and preserves epithelial stem cell characteristics (17). Signal networks in the epithelium and mesenchyme regulate hair morphogenesis and cyclic regeneration. Due to the lack of a reliable model of human scalp HFs, most of the subsequent studies were completed using a mouse model. In cases not explicitly labeled, the laboratory specimens were mice in this part.

##### Wnt Pathway

Wingless proteins initiate HF development, preserve stem cell identities, and guarantee the formation of the hair sheath. The classic Wnt signaling pathway contributes to placode development and stimulates the differentiation of dermal progenitor cells into DCs (18). Specifically, the initial epithelial signal involved in DC development is Wnt10a/b, and Wnt5a is a secondary epithelial signal responsible for the descent of HF into the dermis (19, 20). Although the Wnt/β-catenin and EdaA1/NF-κB pathways both mediate placode formation, Wnt/β-catenin serves as the first and most critical signal related to HF morphogenesis (7). Interestingly, the absence of Lef1, a β-catenin-related molecule, results in structural and functional deficits in mutant mouse glands, teeth, and hair, revealing the central role of the Wnt pathway in skin appendages (21).

During the hair cycle, the hair matrix expresses Wnt3a and Wnt10b, and DP responds to Wnt pathway signals to activate HF epithelial cells (3, 20, 22). Matrix proliferation requires epithelial Wnt/β-catenin; inhibiting β-catenin or exogenously adding Dickkopf (Dkk) can inhibit matrix growth and anagen processing (23). Ectodysplasin A (Eda) indirectly inhibits the Wnt signaling pathway by targeting Dkk; Eda deficiency results in malformed hairs, whereas its overexpression impairs HF periodic renewal (7, 8, 23). Simultaneously, Wnt signaling controls the activity of hair sheath-specific signals, showing its function in HFSC-specific differentiation (24, 25). Furthermore, epithelial Wnt signaling is involved in DP-inducible properties, and experiments found that the addition of Wnt3a rebuilt the inducibility of DP that has been lost *in vitro* in both mice and human tissues (22, 26).

##### BMP Pathway

Bone morphogenic proteins (BMPs) are members of the TGF superfamily and are involved in organ morphogenesis. The human epidermis contains BMPR1A and BMP2, while DCs carry BMP4 and Noggin (8, 26). Inhibited BMP results in increased HF volume, and awl hair replaces zig-zag hair (7). Additionally, through a hybrid knockout test, the BMP signaling pathway was proved to be critical for stem cell rejuvenation: BMP/Wnt signaling maintains stem cell homeostasis by balancing the stem cell, EMI, and the HF–subcutaneous adipose tissue interaction (17, 27). By attenuating the inhibitory effect of BMP on the ectodysplasin A receptor (Edar), connective tissue growth factors can form placodes at regular intervals in human hair regeneration (7). All studies validate the importance of BMP signaling in hair morphogenesis.

Bone morphogenic protein signaling is vital for maintaining DP inducibility and hair differentiation. BMP interacts with receptors in human DP and augments DP inducibility, with its specific ablation leading to an inordinate DP feature (7, 26, 27). BMP–Smad signaling is required to maintain the hair sheath, and BMPR1A deficiency reveals an inclination toward severe hair loss and hair graying (8, 24, 28, 29). In addition, Noggin, a BMP2 ligand inhibitor, is involved in the formation of placodes, and it also prompts HFSC regeneration and HF expansion into the dermis in humans and mice (7, 28).

##### SHH Pathway

Sonic hedgehog is a second dermal signaling molecule in the placode and DP during HF morphogenesis (30). Additionally, SHH adjusts the HF polarity and angle of growth. In the hair cycle, the activated secondary hair germ produces SHH to reactivate the matrix, and HF will be blocked in anagen phase III with ablation of SHH (15, 31, 32). Moreover, the SHH downstream target genes and Gli and Ptch induce HFSC mitosis, with Gli2 activating Sox9 to affect the Wnt pathway (7, 33, 34). Collectively, as a typical morphogenetic signal, SHH is positively involved in HFSC proliferation and differentiation.

#### Growth Factors

Epidermal growth factor influences the lungs, mammary glands, small sweat glands, and skin, which is expressed in the HF outer root sheath and differentiated sebaceous glands (10). The EGF family impedes HF morphogenesis, generally manifesting as placode and DC deficits (7). The EGF family also controls hair sheath differentiation and morphology, with TGF-α mutations and deletions producing wavy hairs owing to distortions in the outer and inner root sheaths (2, 8).

The fibroblast growth factor is located in the epidermis and DC and is involved in placode formation in most circumstances. Similar to embryonic development, FGFR2b ablation results in abnormalities in the granular layer and skin appendages (2, 35, 36). In signal networks, FGF20 is secreted after Wnt signaling activation in epithelial placodes and promotes DC formation (37). Identifying the function of the FGF family is important due to the various mechanisms that they modulate in the hair cycle and related disorders. FGF5/18, as catagen-promoting factors, can inhibit stem cell growth, accelerating the anagen–catagen transition and adjusting the hair sheath length (3, 15, 38, 39). Similar to FGF7/10, more FGFs operate as transient amplifying cell-activating signals, catalyzing the telogen–anagen transition and HF renewal (8).

Transforming growth factor-beta has anti-proliferation potential for most epithelial cells, including follicular keratinocytes. When HF enters catagen, the epithelial TGF-β/activin signal induces apoptosis (3, 8, 38, 40). As a typical human hair disease-related gene, TGF-β deletion in HFSCs impairs the differentiation of adjacent pigmented stem cells in human follicular keratinocyte cells (41, 42).

Platelet-derived growth factor (PDGF) regulates cell growth and mesenchymal cell division, which remains the first epithelial signal in the placode (43). As a downstream target of SHH, PDGF is secreted by the epidermis, with receptors in the dermal sheath and DP, functioning in an adipose-stimulating manner to enhance HF regeneration (8). In addition, PDGF is secreted by the HF matrix during anagen and has a facilitative effect on the hair germ (8, 44).

Secreted by DP, vascular endothelial growth factor (VEGF) stimulates the expression of VEGFR-2 in human epidermal cells, hence promoting their proliferation, differentiation, and migration (45). Interestingly, VEGF can directly act on DP and promote human HF growth by stimulating local blood vessels during anagen, with bFGF promoting VEGF angiogenesis (8, 46, 47).

#### Cytokines and Chemokines

The TNF family member Eda modulates the induction, morphogenesis, and maintenance of skin appendages such as hair, teeth, and sweat glands (48). In HFs, Eda is present in the placode and the interfollicular epidermis throughout embryonic morphogenesis, and its mutation impairs the construction of guard hair in humans. The Eda cascade operates on the placode, and inadequate Eda results in aberrant appendages such as sparse hair, uneven teeth, and the absence of sweat glands (7).

Attention should be paid to the respective functions of IL family members because of their multifaceted regulatory mechanisms during the hair cycle, and the effect of the inflammatory responsiveness of the IL family in alopecia needs also to be studied. IL-36a aggregates HF rejuvenation, while IL-1β drives the anagen–catagen transition (8, 49). As an immunohistochemical biomarker, IL-1 expresses an HF morphogenesis-dependent localization during embryonic-like HF formation, and IL-1R^+^ keratin-forming cells are expressed in the hair germ and the outer root sheath (30), while the IL-2 receptor may be associated with hair regeneration (50). IL-6/10 is a downstream effector of the JAK–Stat pathway, and the JAK–Stat inhibitor ruxolitinib effectively relieved alopecia areata (12).

#### ECM-Specific Components

Human DP secretes a particular ECM, and epithelial cells selectively adhere to and grow on the surface of the basement membrane material, which jointly controls HF-related gene expression (51, 52). Extracellular collagen has a regulatory effect on maintaining DP growth and properties. Proteoglycans act as cell adhesion molecules, transmembrane signaling molecules, growth factor activators, and macromolecular transporters in the ECM. Persistently, low proteoglycan levels contribute to human pattern hair loss and telogen effluvium (53).

The macromolecular proteins in the ECM contribute to the stabilization of the HF microenvironment. Many specific ECM components can interact with small signaling molecules to maintain homeostasis. Specific proteoglycans in human ECM, such as aggrecan, biglycan, fibronectin, hyaluronic acid, and type I collagen, are pro-proliferative (14). Fibroblasts synthesize versican, which constitutes the ECM of human DP and modulates Wnt signaling in the anagen (44). Three secreted proteins, namely, apolipoprotein-A1, galectin-1, and lumican, are coenriched in the rat embryo dermis and induce HF *de novo* by stimulating IGF and Wnt (54). 6-phosphate chondroitin sulfate proteoglycan and cartilage oligomeric matrix protein are typically expressed in human HF connective tissue with HF periodicity, interacting with BMP and various ECM proteins to stabilize the basement membrane. Overexpression of cartilage oligomeric matrix protein in humans induces connective tissue diseases, rheumatic diseases, and scleroderma, leading to hair graying and patchy hair loss (55). These findings demonstrate that ECM components are significant and irreplaceable for HF EMI and deserve further research.

## EMI in Skin Wound Healing *via* HFSCs

Epidermal stem cells in HF can participate in wound re-epithelialization; for example, peri-wound melanocytes can migrate upwards to locate in the epidermis, and no pigment is present in new-born HF. After HF reconstruction, the interaction between bulge HFSCs or secondary hair germs and DPs proceeds the HF cycle, recovers melanocytes, and produces colored hairs (56). These findings inspired us to consider the association between the stem cell populations in HFs and cutaneous healing.

### Overview of Wound Healing

**Figure 2:**
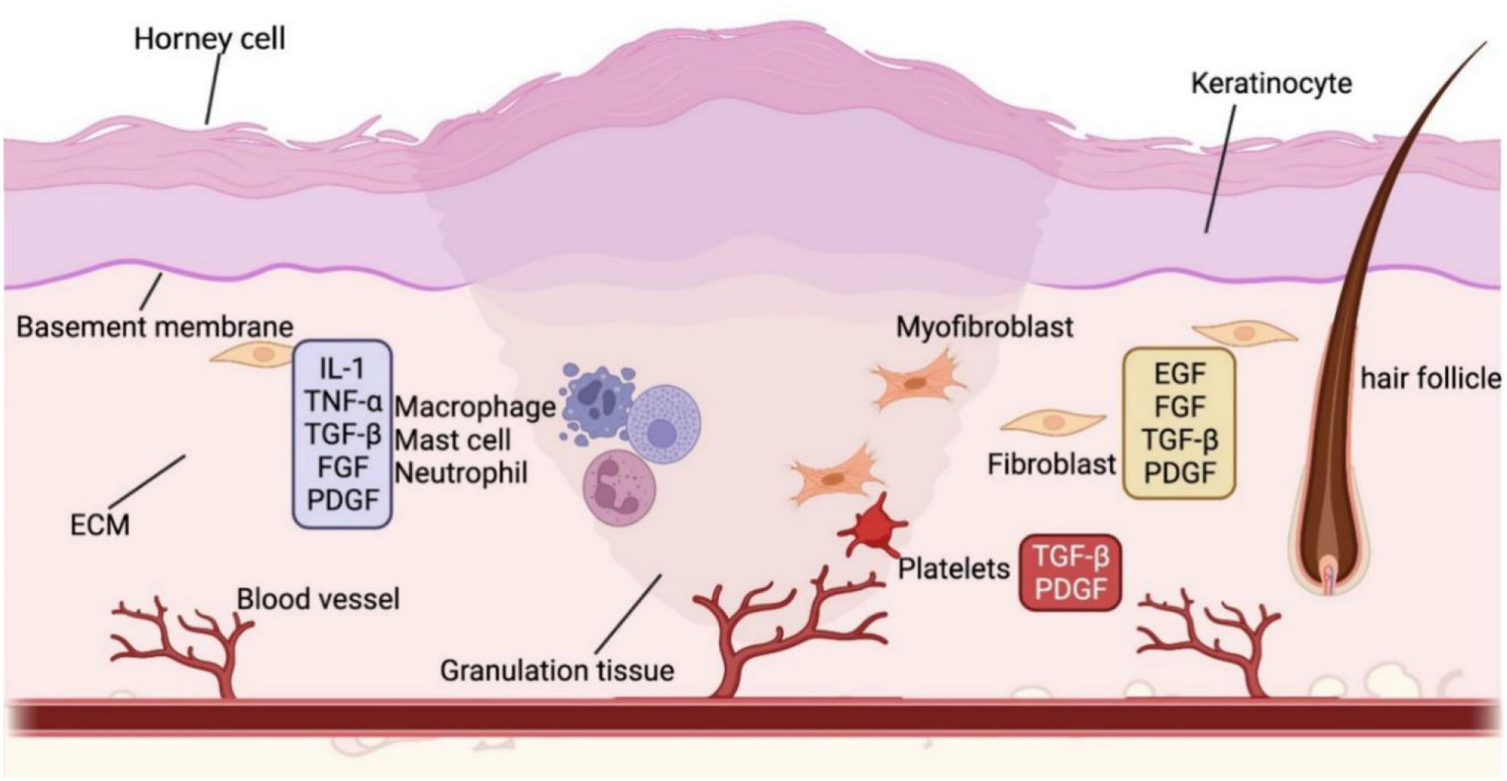
The wound healing process. The various colors in the diagram frames indicate that the molecules in the frame are released by different cells. For example, blue represents inflammatory cells, yellow stands for fibroblasts, and red refers to platelets. Wound healing is a complex biological process. In the wound bed, inflammatory cells proliferate and recruit other required cells in the commence. As the inflammation subsides, endothelial cells in the wound bed increase, followed by the production of granulation tissue and wound site re-epithelialization. The wound contracts and ultimately forms a scar when the extracellular matrix is reconstructed with diminished cell counts. BMP, bone morphogenetic protein; DC, dermal condensate; DP, dermal papilla; Eda, ectodysplasin A; DKK, Dickkopf; EGF, epidermal growth factor; FGF, fibroblast growth factor; HF, hair follicle; HFSC, hair follicle stem cell; IL, interleukin; SHH, sonic hedgehog; PDGF, platelet-derived growth factor; TGF-β, transforming growth factor β; VEGF, vascular endothelial growth factor; Wnt, wingless/integrated.

In minor human skin wounds, myofibroblast contraction enables the formation of new skin without appendages; in more severe injuries, fibroblast contraction ceases, and scars appear before wound closure (56). At the human wound bed, early ECM forms to provide scaffolds for later cell attachment and development. On this basis, inflammatory cells, blood clots, and platelets are recruited, and damaged epithelial and endothelial cells secrete PDGF and chemokines. Then, inflammatory cells secrete the proinflammatory cytokines IL-1 and IL-6 and tumor necrosis factor-alpha (TNF-α) (Figure 2). Granulation tissue, mainly composed of myofibroblasts, keratinocytes, new capillaries, and type III collagen, drifts to the wound bed and initiates re-epithelialization in the lesion. After building a virgin epidermis, type I collagen gradually replaces type III collagen in the ECM, impairing stretching capacity and eventually resulting in scarred human skin devoid of skin appendages (57, 58).

### HFSCs in Skin Wound Healing

#### HF Epidermal Stem Cells in Postinjury Epidermal Regeneration

The HF consists of the infundibulum, the isthmus, and the inferior part encompassing the bulge and DP (Figure 1B). The bulge and isthmus contain HF progenitor cell populations that assist in skin regeneration. Preclinical and clinical studies show that human wound rehabilitation is faster in locations with a high follicular density, such as the scalp, than in areas with a low follicular concentration, such as the palms of the hands and feet (10). Moreover, HFs can be used as autologous cell-derived grafts to offer a novel alternative treatment for long-term unhealed injuries by boosting re-epithelialization, revascularization, and skin restructuring (6, 10). HFs containing dermal fractions are comparable to split-thickness skin grafts concerning epithelialization rates, wound healing, and scar treatment, rendering HF transplantation clinically applicable to them (10).

The regenerated epidermis contains the basal lamina and HFSCs, which move to the damaged cutis and differentiate into epithelial cells, facilitating epidermal restoration by K15-labeled stem cells in the human HF bulge (56, 59, 60). After the injury, HFSCs upregulate cell migration-related genes and reduce bulge gene expression, eliciting epithelial-like characteristics (61). Additionally, as slow-periodic stem cells, bulge HFSCs can simultaneously produce transient amplifying cells, which migrate to specific sites along the basement membrane to repair emergency damage (62). All of the above studies confirm that HFSCs in the bulge region can undoubtedly contribute to re-epithelialization in the lesion.

Additionally, stem cells in the HF isthmus are pluripotent and self-renewing. Lgr6-positive cells are present in the wound neoplastic basal layer, where they bind to collagen to create a scaffold to support re-epithelialization, hair regrowth, and vascularization (10). Notably, in human skin reconstruction experiments, Lrig1-positive cells produce all epidermal cells that move to wounds and repair interfollicular epidermis (56). Gli1-positive cells are competent for long-term multiplication and differentiation after injury and are subject to external niche signaling (56). The stem cell population in the HF isthmus similarly possesses the potential to evolve into skin epidermal cells.

The outer root sheath serves as a reservoir for epithelial stem cells and gives rise to hair germ and matrix formation (15, 63, 64). Nestin-positive cells, presumably outer root sheath progenitor cells, are present in the bulge and upper outer root sheath during the mid-to-late anagen phase (7). Nestin-positive cells can differentiate into keratinocytes, melanocytes, neurons, glial cells, and smooth muscle cells under certain circumstances. A previous study has proved that the transplantation of nestin-positive cells into the severed sciatic nerve interstitial region stimulates neuronal regeneration and restores neurological function, suggesting their role in the skin appendages and neuronal stem cells (6).

Other epithelium-derived cells in the HF are also involved in skin tissue reconstruction after injury. As human sebaceous gland stem cells, Blimp1 protein-positive cells encourage keratinocyte and sebaceous gland differentiation and promote skin repair by sustaining sweat glands, ducts, and interappendage epithelium (6, 65). Under UV irradiation, melanocyte progenitor cells in the upper bulge migrate upwards to the interfollicular epidermis to become functional epidermal melanocytes, protecting the skin (66, 67).

#### HF Dermal Stem Cells in Skin Regeneration

Hair follicle dermal stem cells are fibroblast progenitors in the dermis. *In vitro*, clones of hfDSCs act as skin-derived precursors, healing the dermis following injury (13, 68). *In vivo*, hfDSCs are disposed to differentiate into dermal sheath and DP, smooth muscle cells, neurons, glial cells, and adipocytes, creating adipose and osteogenic tissue (6). DP is organized in the papillary dermis, hosts pluripotent neural crest stem cells, and is also ready to become neurons, glial cells, smooth muscle cells, and adipocytes (66). Therefore, both human hair dermis and mesenchymal stromal cells can be used for induced pluripotent stem cell therapy to rebuild the overall skin structure and improve skin function (37, 69). Moreover, DP promotes keratinocyte differentiation and rebuilds the ECM during human wound healing while minimizing the risk of fibrosis and avoiding the harmful effects of the diabetic environment on cells, which suggests that DP may be utilized in diabetic foot healing instead of bone marrow and adipose mesenchymal cells (37).

### EMI Signals Modulate Wound Healing

#### Wnt and SHH Signaling Pathways

Wnt signaling, a classical cell proliferation factor, also plays a role in cutaneous wound healing (70). Wnt7a upregulates collagen-I/III in the ECM (71), while increased β-catenin triggers matrix fibrosis and injury healing, as evidenced by fibroblast augmentation at the human wound site (37). Wnt signaling can also mediate melanocyte mobility (6, 72, 73). SHH is a typical epithelial signal, and its downstream signaling factor, Gli1, can respond to skin damage. Gli1-positive cells are triggered by injury to generate stem cells with the capability of long-term proliferation and differentiation, which are crucial for wound healing and skin reconstruction (56, 70, 74).

#### Growth Factors

The EGF family, including EGF and TGF-α, is involved in the control of development and cell renewal. Especially, in skin injury repair, EGF induces ECM composition, cell proliferation, and angiogenesis, as well as epithelium and mesenchyme regeneration (58, 70). Furthermore, increased expression of EGFR prompted the formation of an epidermal keratinized envelope (22).

Fibroblast growth factor advances wound healing by improving ECM composition, fibroblast proliferation, keratinocyte migration, and angiogenesis. The above properties qualify FGF as an indispensable signal for granulopoiesis, re-epithelialization, and tissue remodeling (58, 70). In many cases, FGF nourishes the wound area by suppressing collagen synthesis, lowering the density of connective tissue surrounding the DP, and increasing collagen vascular penetration (75). Before completing re-epithelialization in the lesion, T cells produce FGF9, demonstrating that immune cells and skin fibroblasts interact at the injury site to promote wound healing and HF regeneration (56, 57). Significantly, if there is a lack of FGF-9-expressing T cells in the dermis, HFs fail to form in human skin after scarring (60). Moreover, FGF7/10 triggers wound re-epithelialization and angiogenesis by activating keratinocyte mitogenic activity and VEGF secretion in endothelial cells, respectively (57). FGF7/10 deficiency impairs the proper interaction of T cells with keratinocytes, resulting in lower keratinocyte mobilization and limiting injury recovery (36).

In wound healing, TGF-β recruits neutrophils and macrophages, mediates ECM deposition, promotes angiogenesis, and enhances epithelial cell migration (70). TGF-β and PDGF are released by platelets to facilitate fibroblast proliferation and migration and recruit immune cells to build granulation tissue (58, 70). The TGF-β family also regulates the degree of fibrosis in wounds, affecting scarring and the aesthetic properties of living tissue. In adult skin injuries, Wnt3 upregulates the profibrotic factor TGF-β1/2 *via* Smad2, but injecting recombinant human TGF-β3 into the embryo can minimize scarring (37, 57, 76). Fibromodulin-deficient mice show elevated TGF-β3 with reduced fibroblast mobility and delayed cut closure, indicating that TGF-β3 exhibits an anti-migratory effect in the early stages and an anti-fibrosis function in the late phase of wound healing (57).

As a representative epithelial signal, PDGF improves wound healing by recruiting neutrophils and macrophages, activating fibroblasts, recruiting vascular smooth muscle cells, and elevating IGF-1 to increase matrix metalloproteinase expression in the ECM (43, 57, 58, 70). For clinical application, human becaplermin/PDGF-BB, a wound healing factor, is the only FDA-approved profibrotic growth factor used to treat chronic healing failure (57, 68, 77).

Dermal papilla-secreted VEGF promotes epidermal cell migration and suppresses collagen production, making it a crucial growth factor for revascularization after tissue destruction (70, 75). VEGF is a significant growth factor that impacts revascularization and scarring. FGF-2 and VEGF accelerate wound healing in patients with neck dissection (78). A broad difference exists in ECM VEGF in embryonic and adult wounds, while VEGF expression is lower in adult wounds and more prone to forming scars (37, 57). In addition, VEGFR-2 influences the vascularization of keratinocytes in skin appendages such as HFs, sebaceous glands, and sweat glands (45).

#### Cytokines and Chemokines

The proinflammatory cytokines IL-1α, IL-1β, and IL-6 boost fibroblast and keratinocyte multiplication and recruit neutrophils, thus playing an essential role in forming granulation tissue (70). Nevertheless, in the late stages of inflammation, M2 macrophages generate the anti-inflammatory cytokine IL-10, which has antifibrotic properties and whose absence results in scarring, suggesting the potential function of ILs in skincare (57, 58).

#### ECM-Specific Components

Macromolecular components in the ECM play a significant role in human skin wound healing. For instance, high-molecular-weight hyaluronic acid is abundant in embryonic wounds and is beneficial for fibroblast migration, whereas its reduction leads to scarring (37, 79). During human skin damage, type III and type I collagen ratios are approximated to those in the embryo and are accompanied by increased hyaluronic acid. Theoretically, adding hyaluronic acid and collagen content at the wound site can proceed with embryonic wound healing and reduce fibrosis and scarring (9, 80). Decorin and lumican are rich in leucine and engage in collagen assembly, showing antifibrotic activity in adult human skin (37, 44, 57, 81). Fibromodulin is highly expressed in embryonic wounds, reduces fibrosis, and enhances fibroblast contraction by inhibiting TGF-β1. Elastin is responsible for the elasticity of connective tissue, and its deficiency influences the skin's nature (57). Moreover, the interaction of fibronectin and tenascin in sheep's wounds promotes ECM deposition, allowing cells to connect to and migrate within the matrix and advancing re-epithelialization (37). In summary, a collection of ECM components are involved in wound healing through their interactions and associations with adjacent cells (Table 1).

**Table 1:** Signals in hair follicle regeneration and wound healing.

**Signal factor**	**HF formation**	**HF cyclic regeneration**	**Wound healing**	**Research model**	**Reference**
Wnt/β-catenin	Placode and DC formation	Facilitate HF growth and differentiation	Activate epithelial cell proliferation	Mouse and human	(18, 20, 24, 70)
BMP	Negative for placode formation; Noggin is involved in placode development	Enhancing DP induction and maintaining HFSC quiescence; Noggin promotes HFSC regeneration	Conversion of fibroblasts into adipocytes	Mouse and human	(7, 8, 17, 27, 89)
SHH	Placode growth and acts as the second epithelial signal to epithelial cell multiplication	Activate second hair germ to initiate HF periodic regeneration	Favorable to epidermal recovery and regeneration	Mouse	(7, 74)
EGF family	Delayed placode and absent DC	Hair sheath differentiation and morphology	Extracellular matrix composition and epithelial cornification	Mouse	(2, 7, 22, 70)
FGF family	Placode and DC development	Control the length of the telogen phase	Granulation tissue formation, re-epithelialization and tissue remodeling	Mouse and human	(8, 37, 70)
TGF-β	Inhibiting epithelial cell proliferation and placode formation	Induce catagen	Extracellular matrix deposition and epithelial cell migration	Mouse	(38, 65, 70)
PDGF	Acting as the first epithelial signal to shape DC	Enhance HF regeneration	Speed up wound healing	Mouse and human	(7, 8, 57)
VEGF	Epidermal cell multiplication and migration	Maintain the anagen	Angiogenesis and keratinocyte migration	Mouse and human	(8, 45)
IL family	IL-36 promotes HF development	IL-1b triggers catagen	Recruit immune cells and promote fibroblast and keratinocyte multiplication	Mouse	(8, 49, 70)
Eda/Edar	Placode generation	Induce anagen	Promote epithelial wound healing	Mouse and human	(8, 90, 91)
**ECM specific components**	Stable basement membrane	Some proteoglycans are pro-proliferative	Regulate pro-regenerative matrix and wound fibrillation	Mice, human and sheep	(14, 57, 81)

*Epithelial–mesenchymal interaction is essential for HF neogenesis, HF cycle, and skin tissue regeneration. Wnt signaling in the epidermis initiates the placode, and the hierarchies and connections of known signals and pathways govern the development of placode and dermal cohesions, HF downstaging, and HF regeneration. While in the HF cycle, the initial signal arises in DP, and the normal functioning of hair growth is ensured by multiple positive and negative regulators between the matrix and the DP in an autocrine fashion to activate the HFSC or maintain its quiescent state. Each signal acts as a regulator involved in the fluctuation of the HFSC state and the expression of other pathways (Wnt, BMP, SHH, growth factors, etc.), forming a network in the HF cycle. Also, HFSC contributes to re-epithelialization at the wound site. Changes in cell type, growth factors, and extracellular matrix components exist within the injured tissue, accounting for matrix deposition, wound healing, and scar formation. BMP, bone morphogenetic protein; DC, dermal condensate; DP, dermal papilla; Eda, ectodysplasin A; DKK, Dickkopf; EGF, epidermal growth factor; FGF, fibroblast growth factor; HF, hair follicle; HFSC, hair follicle stem cell; IL, interleukin; PDGF, platelet-derived growth factor; SHH, sonic hedgehog; TGF-β, transforming growth factor β; VEGF, vascular endothelial growth factor; Wnt, wingless/integrated*.

## Discussion

Epithelial–mesenchymal interaction is a wide-ranging biological activity involving hair, teeth, and mammary gland morphogenesis. In the past decade, significant progress has been made in the signaling involved in HF regeneration, hair cycle, and wound healing, making the HF an excellent model for investigating EMI. Communication of soluble factors, regulation of key pathways, and transduction of ECM signals all participate in HFSC-regulated EMI in HF tissue. Multiple signallings (Wnt, BMP, SHH, growth factors, etc.) regulate HFSC status and undergo periodic content fluctuations during the HF cycle. Moreover, HFSC can participate in both neo-epithelial tissue and neo-mesenchyme at the wound site, contributing to re-epithelialization. Current skin transplantation promotes cutaneous repair but fails to form intact and functional skin appendages, and common skin grafts are not desirable for healing large burns due to the increased risk of infection and donor region skin loss (10). Successful repair of injuries in the epidermal basal layer and skin appendages (HFs and glands) requires mesenchymal cells, including DP. Therefore, an improved understanding of the role of EMI and HFSC function in follicle regeneration and skin repair may provide a promising option for long-term unhealed wounds (82).

Since human organ culture models are time-consuming and the *in vitro* culture conditions are demanding, most of the studies on HF EMI are currently performed using murine follicular cycle models. However, the mouse model differs from human skin in terms of stem cell niche and skin contractility and is not entirely predictive of all clinical outcomes. Moreover, murine hair is densely distributed on the body surface, while HFs are sparsely distributed on human skin, suggesting that the beneficial effect of HFs on skin healing may be more evident in mouse wounds than in human wound healing. Thus, when replicating mouse experiments on human models, the translational study should be improved to focus on the dissemination differences of skin appendages, biomechanics, and transdermal absorption efficiency (83).

Hair follicle regeneration relies on HFSCs, thus understanding the signals in controlling survival, and death of HFSCs will update clinical guidelines for hair loss and other skin diseases. Notably, human HFs are the predilection sites of some basal cell carcinomas and pilomatrixoma, suggesting a link between human HF cells and skin cancers (84). Zhang found that stem cell damage in DP led to hair loss and tumor formation. In contrast, targeted destruction of Mx progenitors or precursors induced transient hair loss and HF morphological damage without tumorigenesis (24). Growth factors provide further evidence in HF EMI that can exert tumor-suppressive effects in other organs. In direct support of this notion, FGFR2b is associated with tumorigenesis and prognosis in migratory cell carcinoma, salivary gland carcinoma, and prostate cancer, but no significant correlation was observed in skin models (85–87). FGF7/10 can also induce cancer by affecting inflammatory cell properties *via* inflammatory responsiveness (88). Therefore, a controlled dose or alteration of the nature of these tumor-related molecules is vital to ensure that they can induce follicle formation without producing harmful side effects.

Despite recent striking advances in HFSCs, the parallel comparison between HFs and other cell populations still needs further studies. Rendl found that discrepancies in molecular signatures between DP and matrix were similar to those during the separation of neural and non-neural ectoderm in vertebrates (42). Similarly, Taylor compared corneal stem cells and HFSCs and revealed commonalities in slow-cycling, proliferative potential, storage patterns, microenvironment, and tumor relevance (62). Thus, EMI similarities between HF and other organs may help us further probe the role of HFSC in skin regeneration and tumorigenesis.

Clinical trials have shown that HFs can be used as dermal grafts to treat chronic wounds and minor burns, but no clinical studies have used HF transplantation to treat large defects such as extensive burns. The scalp is an excellent donor site for skin grafts, with the advantage of faster growth and re-harvesting ability. Given the critical role of EMI in the majority of hair physiology, further studies are needed in alopecia treatments, and HF progenitor cell transplantation may bring us solutions to some of the concerns about skin grafting to provide future therapeutic applications.

## Author Contributions

M-QM gathered the information and wrote the original draft. JJ is responsible for funding acquisition and modification. All authors contributed to the study, participated in resources, wrote the review, edited, and approved the submitted version.

## Funding

This study was supported by the National Natural Science Foundation of China (Nos. 81972955 and 81972959).

## Conflict of Interest

The authors declare that the research was conducted in the absence of any commercial or financial relationships that could be construed as a potential conflict of interest.

## Publisher's Note

All claims expressed in this article are solely those of the authors and do not necessarily represent those of their affiliated organizations, or those of the publisher, the editors and the reviewers. Any product that may be evaluated in this article, or claim that may be made by its manufacturer, is not guaranteed or endorsed by the publisher.
